# The Coproduced Youth Priorities Project: Australian Youth Priorities for Mental Health and Substance Use Prevention Research

**DOI:** 10.1111/hex.70274

**Published:** 2025-04-23

**Authors:** Kate Ross, Jessica Houston, Emma Barrett, Felicity Duong, Tanya Dearle, Smrithi Ravindra, Cheryl Ou, Kirsty Rowlinson, Marlee Bower, Louise Birrell, Katrina Prior, Lucinda Grummitt, Chloe Conroy, Anna Grager, Maree Teesson, Catherine Chapman

**Affiliations:** ^1^ The Matilda Centre for Research in Mental Health and Substance Use, Faculty of Medicine and Health The University of Sydney Sydney New South Wales Australia; ^2^ Youth Advisory Board, The Matilda Centre for Research in Mental Health and Substance Use and PREMISE NHMRC Centre of Research Excellence in Prevention and Early Intervention in Mental Illness and Substance Use Sydney New South Wales Australia

**Keywords:** coproduction, mental health, prevention, priority setting, research, substance use, youth

## Abstract

**Background:**

New approaches to mental health and substance use prevention, that bridge research and diagnostic silos are urgently needed to address rising trends in mental illness amongst young people. Engaging diverse stakeholders, including young people, in setting research priorities could aid the development of innovative responses, enhance research and improve translation. While previous activities have identified priorities for Australian mental health and substance use prevention research, none have centred young people as a primary stakeholder. The current study is a critical next step to understand youth perspectives.

**Methods:**

This Youth Priorities Project was coproduced with members of The Matilda Centre and PREMISE NHMRC Centre of Research Excellence Youth Advisory Board. The project involved three iterative stages: (1) an online survey; (2) consultations via focus groups and (3) a final consensus workshop to summarise key recommendations for principles, priorities and actions.

**Results:**

A diverse group of young people aged 16–25 were recruited for Stage 1 (*n* = 653), Stage 2 (*n* = 7) and Stage 3 (*n* = 3). Youth participants emphasised the need for increased investment in prevention research targeting a broad range of social determinants and health behaviours and their intersecting effects on youth mental ill health. There were strong calls to government to address economic drivers of mental health, to increase timely access to subsidised mental health support, and to better equip schools to support student wellbeing and mental health.

**Conclusion:**

Young people had valuable and unique insights on how research and policy responses regarding youth mental health and substance use could be improved.

**Public Contribution:**

A coproduction Research Team (CPRT) was established, including two youth researchers who guided all stages of the research from conceptualisation, ethics approval, survey and study design, analysis and write‐up. A Youth Advisory Board was also consulted.

## 1 Introduction

Globally, the rates of depression and anxiety in young people have increased among recent cohorts [1]. Almost half of young people (48.4%) who develop a mental disorder experience the onset of the first disorder before they turn 18, and two in three (62.5%) before the age of 25 [2]. The impacts of mental and substance use disorders on young people and families are considerable and costly for communities in Australia and around the world [3, 4, 5]. The COVID‐19 pandemic has had disproportionate and unprecedented impacts on young people, exacerbating prevailing inequalities and weaknesses in our education, social service and mental health systems [6]. New approaches to mental health and substance use prevention, that bridge traditional research and diagnostic silos, are therefore urgently required to respond to complex challenges and reverse the rising trends in mental illness amongst young people.

Mental and substance use disorders contribute to 15% of the total disease burden in Australia [7]. However, the proportion of funding allocated to mental health provided by the country's two largest health and medical research organisations, the Medical Research Future Fund (MRFF) and the National Health and Medical Research Council (NHMRC) is substantially lower – 7.4%; 11.5% [8]. The Million Minds Mental Health Research Mission recently invested $125 million AUD over 10 years from 2018/2019 to support research into the causes of mental illness and psychological distress, and the best early intervention, prevention and treatment strategies [9]. It is critically important that this and future funding aligns with the needs and priorities of people who experience these conditions, including young people [10].

There is an increased expectation worldwide of involving people with lived experience in determining priorities for investment and action in mental health research with Australian government agencies [10, 11] and researchers emphasising the significance of involving community stakeholders in priority setting [12, 13, 14, 15, 16, 17]. Consumer and lived experience participation in setting research priorities improves the relevance and impact of research and reduces ‘research waste’ [18, 19, 20, 21]. Seeking input from young people and people with lived experience in setting research priorities could help identify gaps to address the full spectrum of issues that impact youth mental health and substance use [20].

While significant international priority‐setting activities for mental health and substance use research have been undertaken [22, 23], only a few involve youth stakeholders [24, 25, 26, 27]. Likewise, while several activities have been undertaken to set priorities for mental health research in Australia [28, 29, 30, 31], to date, no Australian mental health prevention research priority setting activity has sought to understand the perspectives of young people specifically. Young people are in a unique position to guide and contribute to prevention research and they should be involved in all stages of the research process, including in setting the agenda for research priorities [32].

### 1.1 Aims

The current study aimed to understand the perspectives of Australian young people (aged 16–25 years) on funding priorities for youth mental health and substance use prevention research. It also sought feedback on what immediate support governments can provide to reduce the mental health impacts of COVID‐19 among young people. It represents one of the largest international studies of this kind to date, and the first in Australia.

## 2 Methods

The YPP was coproduced with members of the Matilda Centre and PREMISE NHMRC Centre of Research Excellence Youth Advisory Board (YAB) [32]. Our approach was informed by key guidelines and literature on meaningful consumer/youth involvement, as well as coproduction [10, 11, 32, 33, 34, 35, 36] and we built upon previous Australian priority setting activities [28, 29]. To lead every stage of the YPP, a coproduction Research Team (CPRT) was established, including two researchers, three co‐ordinators and two members of the YAB. Throughout the project, CPRT members engaged in open dialogue to co‐reflect on our approach to coproduction. At the conclusion of the project, a series of meetings were held with the CPRT to formally assess both the approach and impacts of involving young people in coproducing each stage of the project. Responses were recorded and tabulated and reviewed by all members of the CPRT (Table 1).

**Table 1:** Approach and impacts of involving young people.

Activity	CPRT	Youth CPRT	YAB	Impact
Secure project funding	★			Protected funding for youth involvement ensured safe and equal partnership.
Set agenda for project	★	★	★	Youth CPRT members report that their involvement in setting the agenda for the project gave them a sense of ownership from the outset, making the YAB & YCPRT teams feel heard.
Provide mentoring in research skills	★			Youth CPRT members report that the research skills and knowledge attained are relevant for their current and future careers.
Define the aim of the project and agree on team roles and responsibilities	★	★		Early conversations about the aim of the project and clearly defining roles and responsibilities helped to ensure a positive coproduction partnership. Designing the project as a group, provided the foundations of codesign and increased research skills and confidence.
Undertake management of the project, e.g. administer survey and payment of participants	★			At the outset, it was agreed that project management tasks would be undertaken by a professional staff member as they had the most time capacity. This allowed Youth CPRT members to focus their attention on research tasks according to their interest and time they had available to commit.
Draft ethics application	★	★		Youth involvement in the ethics application improved the language and acceptability of participant materials and the accessibility and inclusivity of the survey and focus groups. Youth CPRT members gained new research skills.
Develop research design/protocol	★	★	★	Coproducing the research protocol gave the YAB and Youth CPRT members a sense of ownership of the project and ensured that youth voices were heard in the design. The skills developed also increased Youth CRPT members confidence to contribute to the Centre's research activities more broadly.
Develop study recruitment materials	★	★	★	Youth involvement in the development of recruitment materials improved the relevance and acceptability of the materials.
Recruit participants	★	★	★	Leveraging of Youth networks led to reaching and exceeding recruitment goals.
Advise on environment/contextual factors for participant interactions	★	★	★	Youth contributions helped reduce potential barriers to participation in priority setting. Critically, youth team members identified digital access as a barrier for Stage 1, so they designed Stage 2 to provide an opportunity for young people to participate in focus groups who may not have been captured in Stage 1.
Develop data collection instrument(s) (survey, interview guide)	★	★	★	Youth involvement in coproducing the data collection instruments including the survey questions and focus group guide helped improve their appropriateness and relevance for young people.
Draft ethics modification	★	★		Following the completion of Stage 1, Youth CPRT members reviewed the study design and made critical recommendations for an ethics modification, including the addition of Stages 2‐3. This youth informed and iterative approach to designing the study ensured that each stage was guided by youth input.
Facilitate focus groups		★	★	Youth facilitation of the focus groups helped addressed the barrier of power dynamics typically seen between researchers and young people.
Analyse and interpret data	★	★		Youth team members provided practical insights to inform data analysis, helping to improve the relevance of findings for young people.
Develop final list of principles, priorities and actions		★	★	The development of the final list of principles, priorities and actions was entirely led by youth CPRT and YAB members ensuring that the voices of young people were foregrounded in the final recommendations of the study.
Present findings to stakeholders	★	★	★	Youth CPRT and YAB members were empowered to communicate the research findings to stakeholders and advocate for their recommendations to be put into action.
Present at academic conferences	★	★	★	Youth CPRT and YAB members contributed to abstract submissions and presented project findings at key academic national and international academic conferences ensuring that the findings were communicated appropriately.
Write journal manuscripts and final reports	★	★		Youth CPRT have co‐authored all manuscripts to communicate the findings of this study, helping to improve the publications and aiding them to develop research skills and increased confidence in writing for different audiences.
Produce recommendations for action based on research	★	★	★	Youth CPRT and YAB members showed leadership in developing a plan to advocate for action to support the recommendations increasing the likelihood of relevance and uptake.

The research was conducted in three iterative stages:

1. An anonymous online cross‐sectional mixed‐methods survey of young Australians aged 16–25 was conducted to identify mental health and substance use priorities. Quantitative data were analysed using descriptive statistics and qualitative data were analysed thematically.
2. Two online community focus groups were run to gather deeper insights on mental health and substance use priorities. It also sought feedback on what immediate support governments can provide, with qualitative data thematically analysed.
3. A structured workshop was conducted with YAB members to reach a consensus on the final recommendations for principles, priorities and actions for youth mental health and substance use prevention research.

This multi‐stage approach to priority setting was selected by youth CPRT members because it allowed for multiple and flexible opportunities for young people to lead and contribute to the process.

Ethical approval was granted by the University of Sydney's Human Ethics Committee (#2022/057), and youth were reimbursed for their contributions.

Further details about the methods, including descriptions of each stage of the project are contained in Supporting Information: Supplementary A.

## 3 Results

### 3.1 Coproduction Research Team and Youth Advisory Board Leadership

The coproduction research team included two researchers, three co‐ordinators (CPRT) and two youth researchers (Youth CPRT) who are also YAB members. All members of the CPRT worked together as equal partners and co‐researchers to design and undertake the study. Other members of the YAB also contributed to the design, conduct and analysis of the study via consultations. One YAB member led the Stage 2 focus groups. Table 1 reports the approach and impacts of involving young people in coproducing each stage of the current project.

### 3.2 Stage 1: Online Survey of Young Australians

#### 3.2.1 Participants

A total of 653 young people completed the survey from April to July 2022 (mean age = 17.8, SD = 2.3). More than half (54.36%) of participants identified as female, and 12.25% identified as nonbinary or other gender. More than two‐fifths (42.11%) of participants identified as bisexual, gay or lesbian and over 40% resided in regional, rural or remote locations. More than three‐ quarters (76.26%) of participants reported lived experience of a mental health condition/s, nearly half (46.55%) reported that they are a carer for a family member or friend with lived experience of a mental health condition (46.55%), and just under a third (31.55%) reported that they are a carer for a family member or friend with lived experience of an alcohol or other drug use issue/s (see Table 2).

**Table 2:** Demographic Characteristics (*n* = 653).

Characteristic	*n*	%
***Experience with mental health conditions***		
Lived experience of mental health condition/s	498	76.26
Carer for a family member or friend who has a lived experience of a mental health condition	304	46.55
Carer for a family member or a friend who has lived experience of an alcohol or other drug use issue/s	206	31.55
Lived experience of an alcohol or other drug use issue/s?	123	18.84
***Gender***		
Female	355	54.2
Male	188	28.7
Nonbinary	80	12.2
Other	23	3.5
Prefer not to say	7	1.1
***Sexual orientation***		
Heterosexual	230	35.1
Homosexual	105	16.0
Bisexual	170	26.0
Other	99	15.1
Don't know	39	6.0
Prefer not to say	10	1.5
***Australian State or Territory***		
New South Wales	252	38.5
Victoria	104	15.9
Queensland	90	13.7
Northern Territory	11	1.7
South Australia	34	5.2
Western Australia	52	7.9
Tasmania	53	8.1
Australian Capital Territory	57	8.7
***Geographic distribution***		
Metropolitan	378	57.7
Regional	208	31.8
Rural	64	9.8

*Note:* Totals vary due to small amounts of missing data for some variables.

#### 3.2.2 Involving Young People in Setting the National Mental Health and Substance Use Agenda

Participants were asked if young people should be involved in setting the national mental health and substance use prevention research and policy agenda. Nearly all 562 participants who responded to this question indicated that they ‘strongly agree’ (67.8%) or ‘agree’ (30.8%) that young people should be involved. One participant stated *how can you help someone if you haven't asked them what they need? You can't. If you want to help young people, ask them what they need.* Another participant implored *not only do we deserve a voice on issues that affect us, we NEED a voice*. Some participants identified the importance of dedicating sufficient resources to ensure that young people are deeply and sustainably engaged as partners, ***…**not as a one‐off consultation to grasp the ‘youth voice’ – actually making a consistent effort*.

#### 3.2.3 Usefulness of Youth Involvement in Setting Research and Policy Priorities

**Figure 1:**
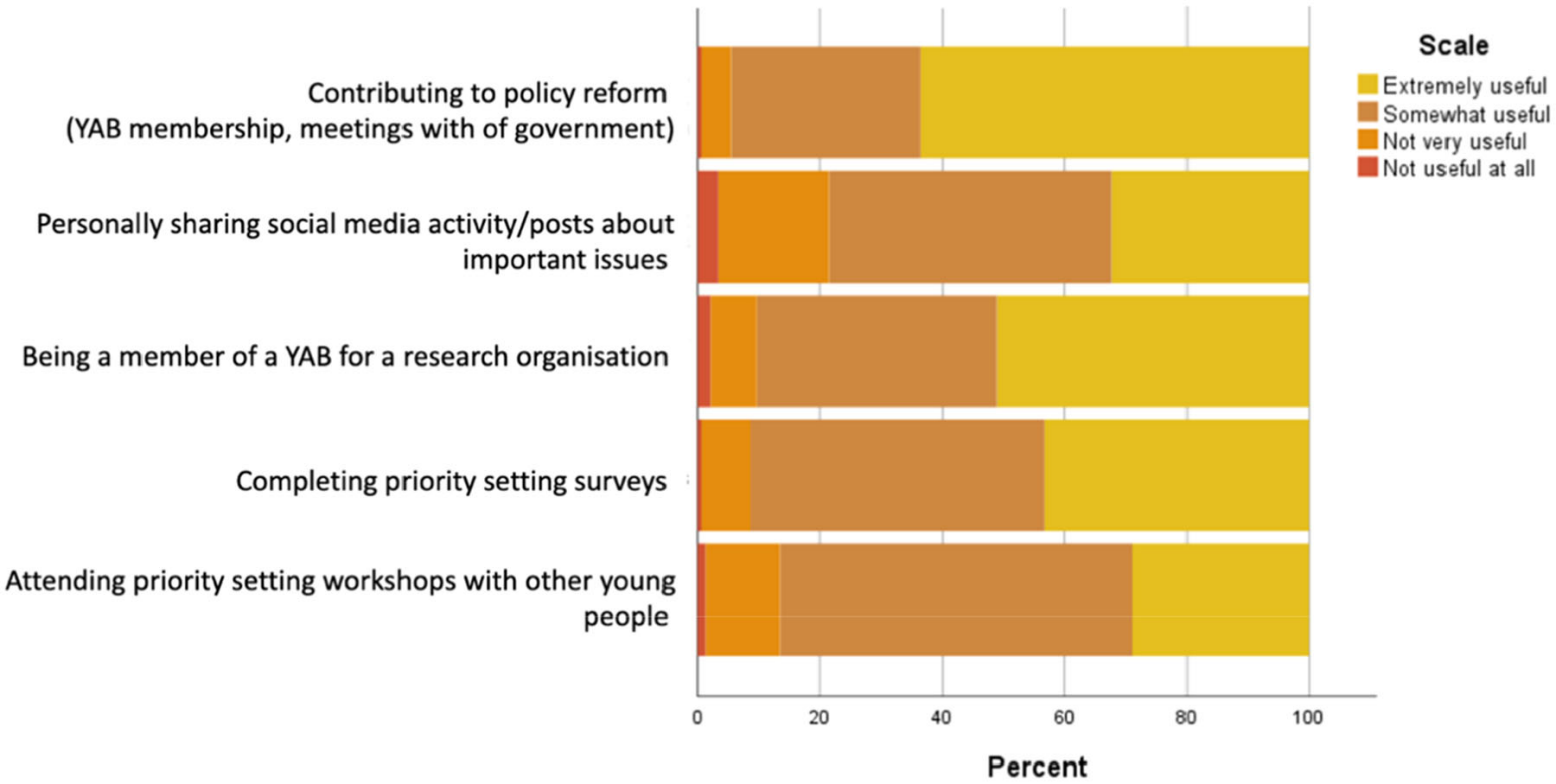
Perceived usefulness of youth involvement in priority setting for mental health and substance use prevention research. *Note:* 339 participants did not respond.

The survey asked participants to indicate the usefulness of youth participation in different priority‐setting activities. As shown in Figure 1, all methods for youth involvement in setting research and policy priorities were considered to be useful. However, one participant noted that all participation options required an individual to be *outgoing, sociable, and [a] brave individual to leave their friendship group to go to a place they don't know with people they don't know’* and they suggested that online engagement may facilitate input form a broader range of young people. Notably, contributing to policy and reform was considered the most useful form of participation.

#### 3.2.4 Investment in Prevention Research

The survey asked participants if they think it's important to increase funding for prevention research. Up to 223 participants (34.1%) provided text comments with their views, with the majority of responses (89.0%) calling for increased investment in prevention research, as *prevention can limit pain and suffering, equip young people with effective self‐care and wellbeing strategies*. Participants who were unsure (5.3%) or did not agree (5.3%) tended to conclude that more research should be going into improving our current mental health systems before introducing new mental health structures as *it is important to identify people at risk and provide preventative measures, however I would like to see more funding go to the ongoing management of mental health issues*.

#### 3.2.5 Priority Populations for Future Research

The survey asked participants to review a list of priority populations for targeting mental health and substance use prevention research. For each population, they were asked to rank its importance on a scale from 1 to 4, where 1 indicates ‘Not important at all’, 2 indicates ‘Somewhat unimportant’, 3 indicates ‘Somewhat important’ and 4 indicates ‘Very important’. A substantial majority of participants who responded to this question (*n* = 276) highlighted particular groups as ‘very important’ priorities for prevention research: individuals who have undergone traumatic or stressful events (e.g., physical or sexual assault, accidents and natural disasters) at 87.3%, individuals facing socioeconomic adversity at 85.3%, Aboriginal and Torres Strait Islander peoples at 77.2%, individuals identifying as lesbian, gay, bisexual, transgender or intersex at 73.75%, and young people (in general) at 70.2%. Feedback from open‐ended responses indicated that many participants were averse to ranking priority populations. Some participants asserted, through open text comments, that every demographic holds significance, and that marginalised or disadvantaged minority groups should all be actively prioritised.

#### 3.2.6 Priority Issues for Future Research

Participants were asked to review a list of 10 broad issues identified by the YAB as negatively impacting youth mental health in Australia (drugs and alcohol, climate change, COVID‐19, unemployment, poverty, intergenerational trauma, unhealthy relationships, isolation and social disconnectedness, lack of accessibility to healthcare in rural and remote settings, social inequality and an ‘other’ option for participants to specify any additional issues). Participants were instructed to select the single issue they believe to be the most important and relevant to young people's mental health. Notably, the highest endorsements by the 208 participants who responded to this question were for isolation and social disconnection, followed by unhealthy relationships, climate change, intergenerational trauma and social inequality. Qualitative responses included feedback that ranking priorities, for example, picking from a list of most important priority populations or social determinants was inappropriate as all populations should be targeted for prevention initiatives.

#### 3.2.7 Health Behaviours

The survey asked participants to rank how important it is for mental health prevention research to consider other health behaviours (e.g. alcohol use, tobacco use, vaping, other drug use, screen time, fruit and vegetable consumption, physical activity, sleep). A majority of the participants who responded to the question (*n* = 254) indicated that it's very important for mental health prevention research to consider other drug use (72.4%), alcohol use (70.1%) and sleep (58.9%). When asked in the survey, ‘Thinking about these behaviours, which is the most important for future mental health prevention research?’ the participant open text responses demonstrated a pushback on studying behaviours in isolation. The following participant accounts unpack the importance of considering this complexity: *These behaviours should not be considered in isolation because they often compound each other and Our experiences are complex and nuanced and cannot be neatly understood in clear‐cut categories, to understand a queer Indigenous person's experiences, you must understand their queer experiences and their Indigenous experiences, and how the two impact each other*.

#### 3.2.8 Social Determinants

**Figure 2:**
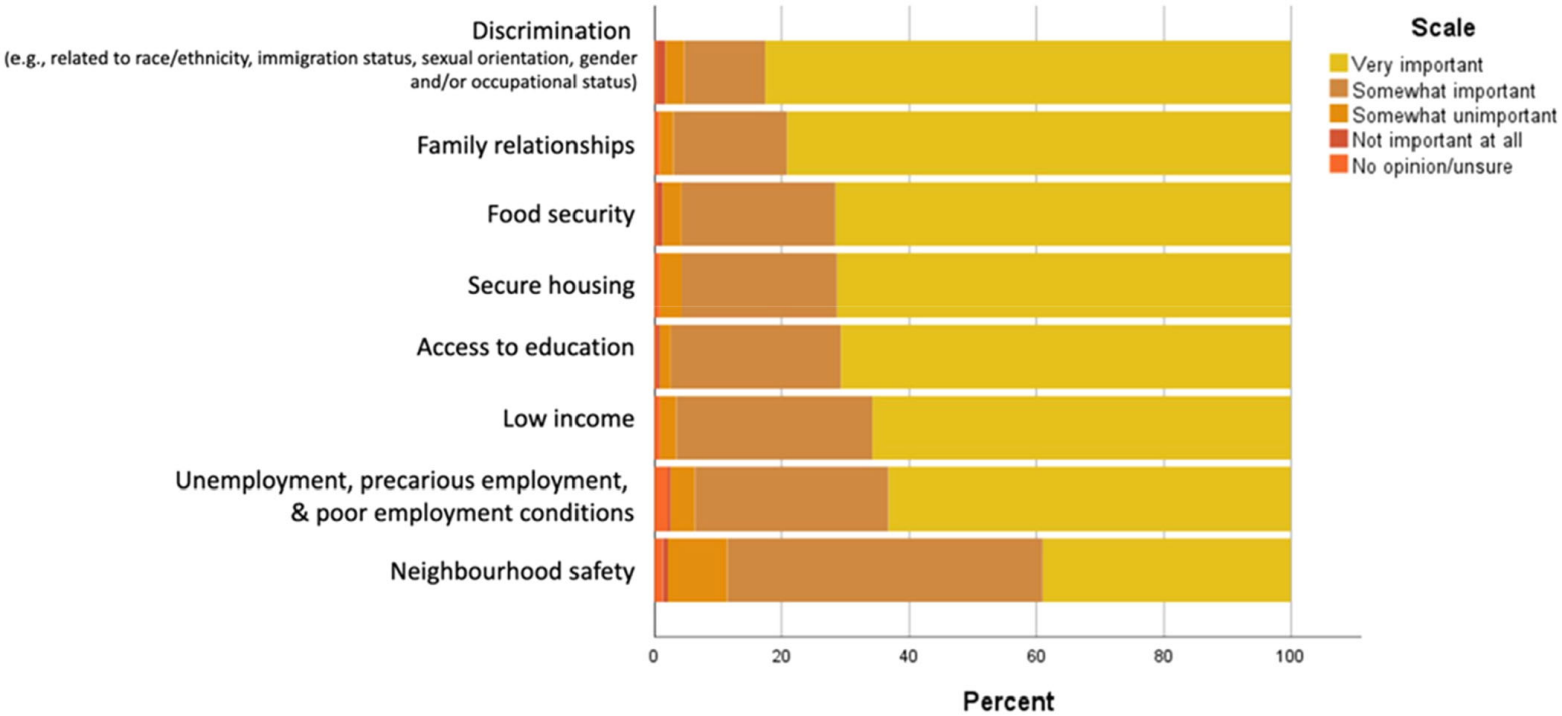
Importance of social determinants for future research. *Note:* 567 participants did not respond.

Participants were presented with a list of eight social determinants of health and were asked to rank how important it is to consider each in future mental health research (see Figure 2). The measures were selected by youth CPRT team members. Participants were then asked how important it is to study the combined impacts of social determinant factors on mental health. The majority (78.88%) of those that responded (*n* = 232) indicated that it's very important that factors are considered together given their intersections.

#### 3.2.9 Actions to Reduce the Mental Health Impacts of COVID‐19

The survey asked participants for their thoughts on what supports governments should provide to reduce mental health impacts of COVID‐19 in young people. 251 participants provided text comments with many calling for improved and subsidised access to high‐quality mental health support and increases to government income support (see Table 3). There were also strong calls for schools to be better equipped to support student wellbeing and mental health. Other suggestions included support for specific groups (e.g., *LGBTQ+, Aboriginal and Torres Strait Islander communities, neurodivergent young people*), improved accessibility of alternate therapy options (e.g., *telehealth, non‐verbal therapy options such as texting*), and community‐led support and support in regional and rural areas.

**Table 3:** Government support to reduce mental health impacts in young people.

	Frequency, *n* (%)
Subsidised Counselling (*e.g., increased Medicare rebate, free counselling services*)	66 (26%)
More mental health resources (*e.g., more psychologists, more informational resources, more services, improved access*)	42 (17%)
Improve school support (*e.g., more school counselling available, safe spaces in school to talk about mental health, emphasis on mental health, less workload and exam pressure*)	35 (14%)1010
Psychoeducation efforts (*e.g., awareness campaigns, school curricula including mental health education, informational resources, mental health advertising*)	27 (11%)
Increased financial support (*e.g., increase income support payments and financial assistance to access education*)	19 ($8)
Increased *mental health* funding efforts (*e.g., funding for research, for mental health organisations, helplines, for more psychologists*)	15 (6%)
Incentivise mental health careers (*e.g., with scholarships, better university programs, better support for mental health workers*)	10 (4%)
Other or unsure	37 (15%)
Total	251

### 3.3 Stage 2: Community Focus Groups to Gather Deeper Insights

Difficulties with recruiting young people led to a decision not to conduct focus groups in Karratha, Western Australia. Two online focus groups were facilitated in Brisbane, Queensland, Australia, in January 2023. The YAB facilitator shared the key findings of the Stage 1 survey and asked participants to discuss whether the findings reflected experiences in their own communities. A total of 7 youth participants aged 16–25 years (4 females, 1 male, 2 nonbinary) participated across the two focus groups. Participants included young people who identify as LGBTQIA+, Aboriginal and/or Torres Strait Islander, having a disability, culturally and linguistically diverse, and having lived experience with mental health and/or substance use issues. Participants resonated with the Stage 1 online survey results and discussed the importance of prevention research to understand and respond to the nonmedical factors that contribute to youth mental health outcomes. Participants called for improved access to high‐quality, timely and Medicare‐subsidised mental health services. Under an intersectional umbrella, three key themes and nine subthemes arose regarding youth priorities for investment in youth mental health and substance use prevention research and actions governments can take to immediately support youth mental health. The three key intersectional themes were (1) social determinants, (2) barriers and enablers, (3) youth leadership and partnership (see Table 4).

**Table 4:** Community focus groups: Key themes.

Interconnected themes	Subtheme	Key quote
Social determinants	Isolation	…*feeling like you are a part of something bigger than yourself and that you have a purpose*
	Trauma	*it's really hard to heal and work on yourself, if you're in an environment where everyone else is hurting*
	Poverty	*It all kind of relates back to that poverty thing*
	Income and social protection	*All Centrelink payments need to be above the poverty line.*
	Education	*The education system is focused too much on grades and stuff while isn't it building life skills, breath and play and kind of developing a sense of who you are, what you're good at*
Barriers and enablers	System reform	*Even with the subsidised rate, you still pay 100 plus dollars per session. No one can afford that.*
	Siloed research and services	*A streamlined way to get into therapy, without having to worry so much about having to refer to like, six different people at once, and being able to afford them at the same time*
Youth partnerships and leadership	Diversity	*So, when they actually interviewed the real people who were part of this study, they were getting what, y'know the right results?*
	Lived experience	*The best research is guided by people with lived experience from the very beginning.*

### 3.4 Stage 3: YAB Consensus Workshop

**Figure 3:**
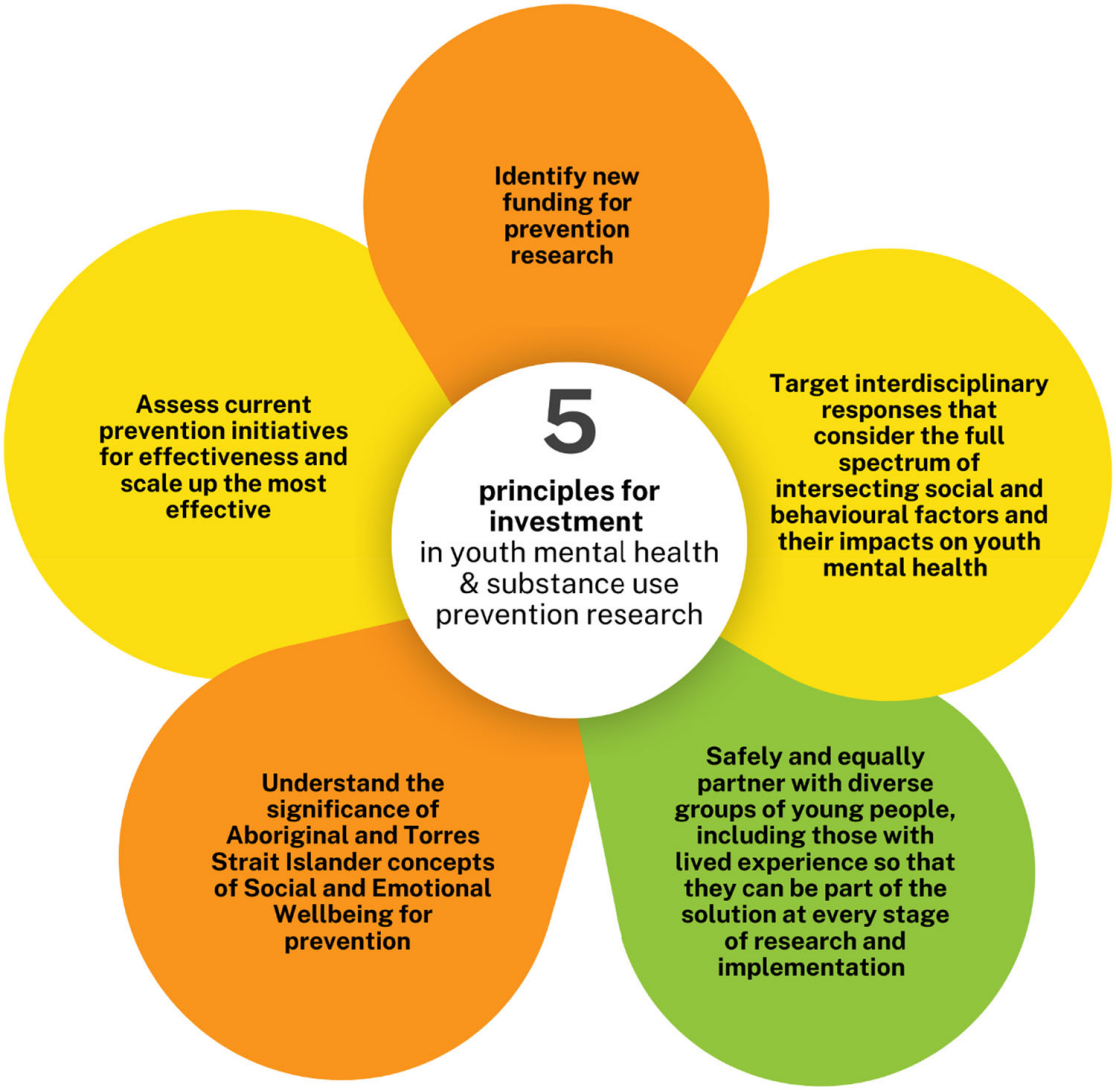
Principles for investment in youth mental health and substance use prevention research.

**Figure 4:**
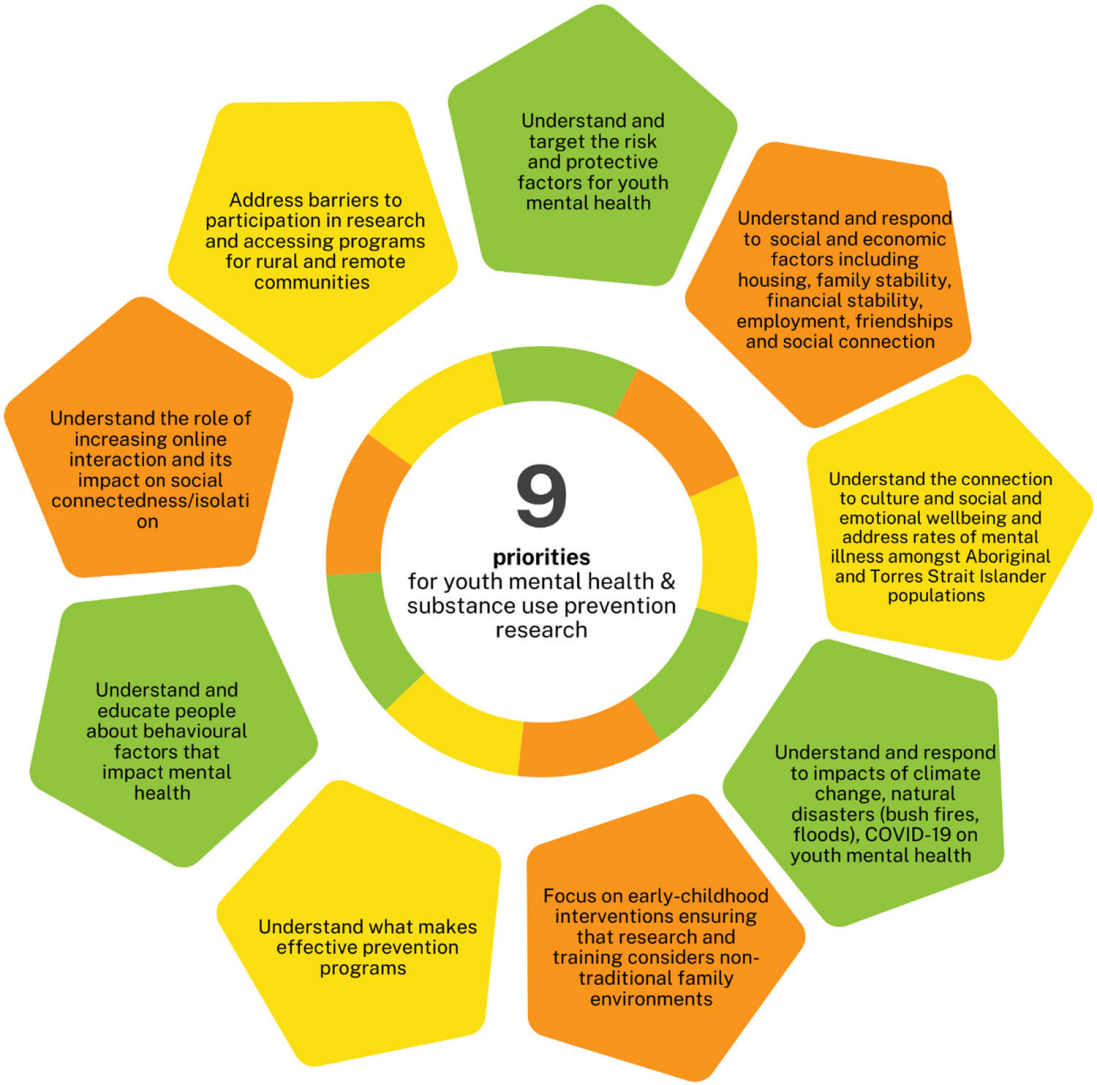
Priorities youth mental health and substance use prevention research.

**Figure 5:**
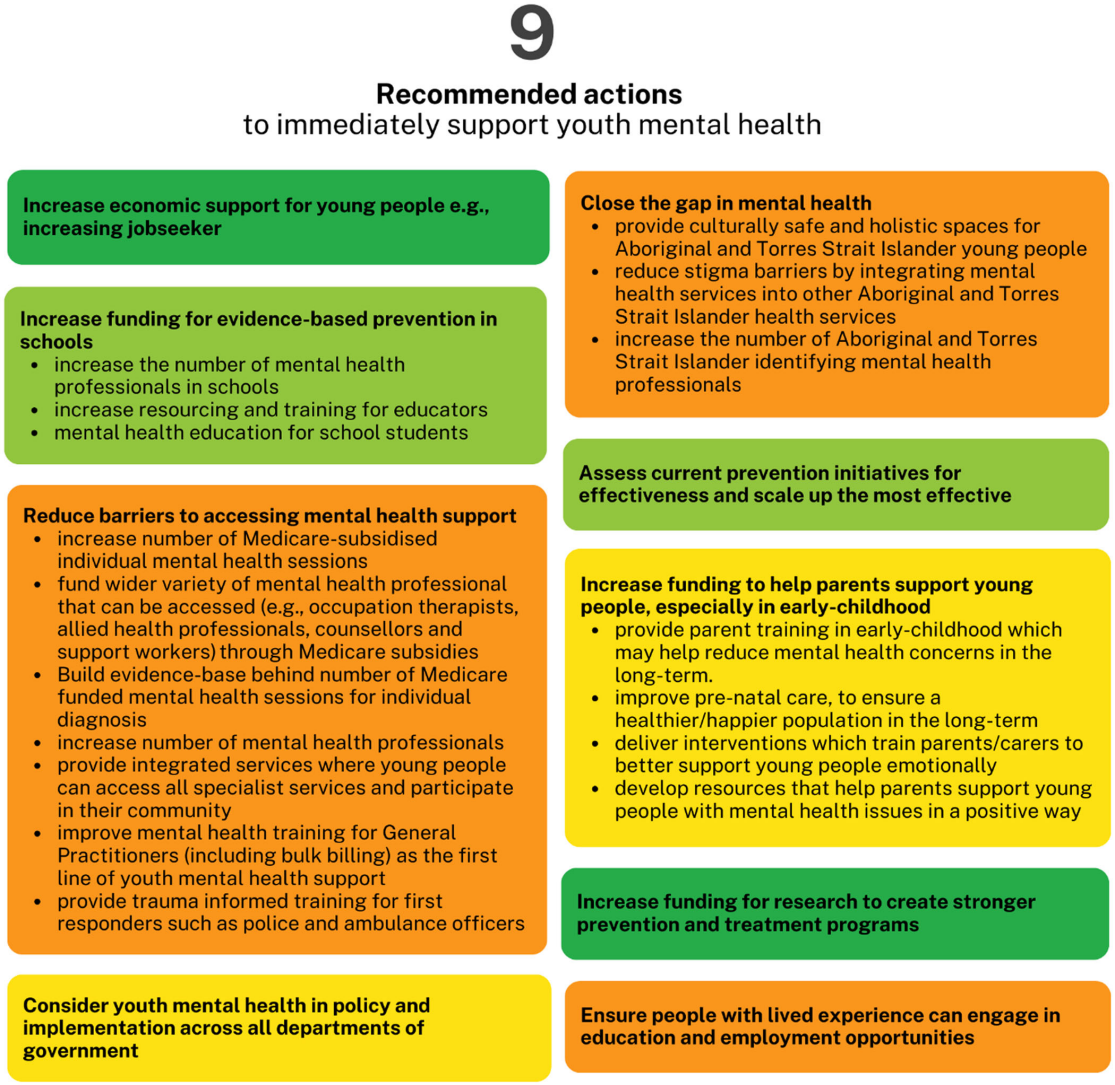
Recommended actions to immediately support youth mental health.

A workshop was led by a youth CPRT member to reach a consensus on final recommendations for principles, priorities and actions. Participants (*n* = 3) analysed and synthesised the input gathered from the 660 young Australians who participated in the earlier Stages of this project. Using the digital tools (https://easyretro.io/) and discourse, consensus was reached on five principles for investment in youth mental health and substance use prevention research (Figure 3), nine priorities for youth mental health and substance prevention research (Figure 4) and made eight recommendations for actions to immediately support youth mental health (Figure 5).

## 4 Discussion

Prevention of youth mental and substance use disorders are a global health priority requiring novel and well‐targeted research responses informed by young people. This study was coproduced with young people and aimed to seek the perspectives of Australian youth aged 16‐25 on funding and research priorities for youth mental health and substance use prevention research. The top issues affecting youth mental health identified in the current study were isolation and social disconnection, followed by unhealthy relationships, climate change, intergenerational trauma and social inequality. Youth participants called for increased investment in prevention research as well as concurrent targeting of a broad range of social determinants and health behaviours and their intersecting impacts on youth mental ill‐health. There were also strong calls for immediate responses to reduce the ongoing mental health impacts of COVID‐19 on young people.

The principle identified in Stage 3 that researchers should safely and equally partner with diverse groups at every stage of research, aligns with previous priority setting activities, which emphasise the value of all types of knowledge, including cultural, experiential, clinical and scientific knowledge, and call for researchers to support codesign/coproduction, especially with people with lived experience and their families, Aboriginal and Torres Strait Islander Peoples and young people [29, 30]. There is evidence that involving young people and people with lived experience in research leads to improved outcomes [32, 37], and the high response rate to the online survey in the current study indicates that a diverse range of young people are motivated to participate in research priority setting activities. Conversely, the lower response rate to participation in the focus groups may indicate that the online survey format is a more effective way to facilitate broad youth participation.

Approaches to research priority setting typically have sought to identify priority populations to target resource allocation and research efforts, leading to the development of a list of research targets [12, 28]. The current study asked participants to rank priorities adapted from previous priority setting activities in the literature and/or developed by the CPRT. However, it is important to note that the online survey questions in the current study asking participants to rank priority populations received low response rates, with most of the qualitative responses indicating that young people felt all populations should be considered equally important. This is interesting and highlights a key difference in perspectives between research funding bodies, researchers and young people regarding the priority setting processes. To address this feedback, the iterative and youth‐led design of Stages 2 and 3 of the YPP avoided asking young people to rank priority populations or issues. Rather, participants were asked to identify key targets overall to facilitate a list of priorities for research. Future youth‐focused research priority‐setting should be coproduced with young people to ensure that survey questions, particularly those involving ranking, are designed in a way that resonates with youth perspectives and encourages meaningful participation.

Participants called for research responses to consider a wide range of social determinants and health behaviours and their intersecting impacts on youth mental ill‐health through interdisciplinary research to inform cross‐government policy prevention responses. This is consistent with the National Mental Health Research Strategy which calls for the adoption of a ‘whole of person and whole of life approach’ to understanding youth mental health [38] and the call from Grummitt et al. for countries to implement a national ‘immunisation schedule’ targeting the social determinants of health across the lifespan with the hope to reduce the demand on mental health treatment services [39]. Youth participants indicated that it's important for prevention research to understand and address a broad range of risk and protective factors, including adverse childhood experiences and social connectedness across the lifespan. Participants made a strong call for increased investment in prevention research. This finding is also supported by the Baker et al. priority setting activity, which called for major funding schemes to increase targeted funding for prevention [29]. Future investment in prevention research could respond to this feedback by focussing on prevention research targeting multiple shared risk factors for mental illness and substance use across all youth demographics.

Participants identified a broad range of actions that governments can take to reduce the mental health impacts of COVID‐19 on young people. There were strong calls for governments to address economic drivers of mental health through increased income support for young people. Consistent with recommendations in the Australian Youth Recovery Plan from Australia's Mental Health Think Tank 2022, young people asked for governments to increase timely access to subsidised mental health support and to better equip schools to support student wellbeing and mental health [6].

This study represents the largest Australian youth‐focused mental health prevention research priority setting activity to date. A diverse range of young people participated with strong representation from gender and sexuality diverse young people, those with lived experience and those living outside of metropolitan areas. As one of the first studies of its kind, this is a considerable strength. Our approach to coproduction was informed by best‐practice codesign guidelines and built upon existing strong and equal partnerships between CPRT members. Youth CPRT members played a critically important leadership role in conceiving and implementing all Stages of the project, ensuring design, recruitment materials and survey were suitable for young people. However, a few limitations need to be noted. The sample used in this study was derived from self‐selected participants and convenience sampling methods which may limit generalisability to the broader population of young Australians. We encountered challenges in recruiting young people to participate in the regional focus groups (Karratha, Western Australia), which led to the decision to only hold focus groups in one metropolitan location (Brisbane, Queensland). This highlights the difficulty of amplifying regional youth voices in research. An online survey format may be a more effective way to facilitate broader youth participation.

## 5 Conclusion

To address the global youth mental health crisis, it is critically important that prevention efforts are efficiently targeted and align with the needs and priorities identified by young people. Young people are uniquely placed to contribute to research and policy responses, and they are highly motivated to do so. Actively involving young people in setting mental health and substance use prevention research priorities can help bridge traditional research and diagnostic silos and develop innovative responses to complex issues. In this study, there was a strong alignment with previous research priority setting studies among other stakeholder groups [29] underscoring calls for increased investment in prevention as well as for the adoption of interdisciplinary responses that consider the full spectrum of intersecting social and behavioural factors and their impacts on youth mental health [37]. A clear point of difference that arose from the youth perspective was a dislike for setting priorities for research based on the importance of one group over another. Whilst this represents a challenge to researchers and funders who are driven to identify targeted funding to priority groups, it highlights the importance of taking a holistic approach to mental health and substance use prevention that considers the impact of intersecting social determinants regardless of the ‘population’ to which individuals belong.

## Author Contributions

**Kate Ross:** writing – review and editing, writing – original draft, project administration, supervision, formal analysis, investigation, methodology, conceptualisation, data curation. **Jessica Houston:** writing – review and editing, writing – original draft, project administration, formal analysis, investigation, methodology, conceptualisation, data curation, validation. **Emma Barrett:** supervision, funding acquisition, methodology, conceptualisation, writing – review and editing, writing – original draft. **Felicity Duong:** writing – review and editing, writing – original draft, project administration, formal analysis, investigation, conceptualisation, methodology. **Tanya Dearle:** conceptualisation, methodology, data curation, investigation, formal analysis, writing – review and editing, writing – original draft. **Smrithi Ravindra:** writing – review and editing, writing – original draft, formal analysis, methodology, conceptualisation, data curation, investigation. **Cheryl Ou:** writing – review and editing, investigation. **Kirsty Rowlinson:** writing – review and editing, formal analysis, data curation, validation. **Marlee Bower:** writing – review and editing. **Louise Birrell:** writing – review and editing. **Katrina Prior:** writing – review and editing. **Lucinda Grummitt:** writing – review and editing. **Chloe Conroy:** writing – review and editing, methodology, conceptualisation. **Anna Grager:** writing – review and editing. **Maree Teesson:** funding acquisition, writing – review and editing. **Catherine Chapman:** funding acquisition, writing – review and editing, supervision, methodology, investigation, writing – original draft.

## Ethics Statement

Ethical approval was granted by the University of Sydney's Human Ethics Committee (#2022/057).

## Conflicts of Interest

We wish to confirm that there are no known conflicts of interest associated with this paper. The Youth Advisory Board is supported by an Australian National Health and Medical Research Council (NHMRC) Centre of Research Excellence Grant (PREMISE; APP11349009). This study was also supported by the NHMRC via Fellowships (APP2026552 to C.C.; APP1195284 to M.T.; APP1195852 to E.B.). The funders had no role in the study design, data collection and analysis, decision to publish or preparation of the manuscript. Ethical approval was granted by the University of Sydney's Human Research Ethics Committee (#2022/057).

## Supporting information

Supplementary_material_A_Methods.

Supplementary_material_B_Stage_1_Online_Survey_Questions.

## Data Availability

The authors have nothing to report.
