# Supplementary material for: The Coproduced Youth Priorities Project: Australian Youth Priorities for Mental Health and Substance Use Prevention Research
Source: Health Expect. 2025 Apr 23;28(3):e70274. doi: 10.1111/hex.70274 (PMC12015976; doi:10.1111/hex.70274)
Supplement: Supplementary file 1 — Supplementary_material_A_Methods. [file HEX-28-e70274-s002.docx]

**The Co-produced Youth Priorities Project – Detailed Methods**

## ***Co-production approach***

A co-production research cycle requires lived experience leadership in co-planning, co-designing, co-conducting and co-reflecting on research^34^. Our approach was informed by key guidelines and literature on meaningful consumer/youth involvement, as well as co-production^10,13,33-36^ and built upon existing strong youth engagement approaches developed with our Youth Advisory Board (YAB)^33^.

**Youth Advisory Board (YAB)**

The YAB was established in 2019 by The Matilda Centre and PREMISE NHMRC Centre of Research Excellence to support the sustainable, productive and safe engagement with young people as partners in our research. The development of the YAB was informed by existing literature on youth mental health and substance use advisory groups, as well as consultations with youth coordinators at organisations with existing youth advisory models.

YAB members have were recruited through a nationally competitive process, and have a personal connection to mental health and/or substance use, whether through their own lived or living experience or that of their family, friends, or community. The YAB members who contributed to the current project, were aged between 18 and 25, represented a diverse cohort in terms of cultural background, gender, neurodiversity, and sexual orientation. The group included participants from metropolitan, rural, and regional areas across Australia.

**Co-production Research Team (CPRT)**

To lead the Youth Priorities Project (YPP), a Co-Production Research Team (CPRT) was established, including two researchers, three co-ordinators and two members of the YAB. Throughout the project, CPRT members engaged in open dialogue to co-reflect on our approach to co-production^34^. At the conclusion of the project, a series of co-reflection meetings were held to formally assess both the approach and impacts of involving young people in co-producing each stage of the project (Table 1). Youth CPRT and YAB members were reimbursed for their time and contributions to the YPP.

The YPP builds on previous work of the YAB^33^ and consisted of three iterative Stages outlined below. This multi-stage approach to priority setting was selected by youth CPRT members because it allowed for multiple and flexible opportunities for young people to lead and contribute to the process.

The research was conducted in three iterative stages:

**Stage 1: Online survey of young Australians**

An anonymous online cross-sectional mixed-methods survey was conducted to understand youth priorities for prevention research in mental health and substance use (see Supplementary Material B). The survey included demographic questions, multiple choice and ranking questions on youth involvement in research, priority populations, co-occurring risk behaviors (e.g., alcohol use, screen time, sleep), and social determinants of mental health (e.g., employment, housing, discrimination). Open-ended questions explored youth opinions on research funding, government support and key issues affecting youth mental health. Survey items were based on previous priority setting work conducted by the YAB and existing literature. Study materials were co-designed by all members of the CPRT and reviewed by YAB members (n=5).

## ***Participants***

Participants eligible for Stage 1 were Australians aged 16-25, recruited via convenience sampling and promotion through study investigator networks and institutional social media channels (Twitter, Instagram, and Facebook). Participants were linked to an online consent form and survey. Upon completion of the survey, participants could consent to enter a prize draw for one of two $250 gift vouchers.

## ***Analysis***

The quantitative data (including demographic details, multiple choice and ranking questions) were descriptively analysed in Excel. Qualitative open text responses were reviewed and analysed using the General Inductive Approach (Thomas, 2016). This involved first a close reading of all qualitative the data by JH and KiR who identified common themes, and collated significant quotes related to these themes.

## **Stage 2: Community focus groups to gather deeper insights**

Two online community consultations were run by a YAB member to gather deeper insights on youth perspectives. This Stage was designed by Youth CPRT members to gather deeper insights from young people. One regional location (Karratha, Western Australia) and one metropolitan location (Brisbane, Queensland) were selected by youth CPRT members as they had connections with these communities. The focus group guide, script, and questions were developed by youth CPRT members, informed by the Stage 1 survey and designed to elicit feedback on the findings from Stage 1 and inform the selection of priorities. Questions were semi-structured, allowing an open-ended conversation between the YAB facilitator and participants. Recruitment materials were co-created with youth CPRT (n=2) members and reviewed by YAB members (n=5). Two research CPRT members attended the focus groups to support facilitation and to scribe. The consultations were audio recorded and transcribed.

## ***Participants***

Participants eligible for Stage 2 were Australians aged 16-25 from communities in Karratha, Western Australia and Brisbane, Queensland. Participants were recruited via convenience sampling through investigator networks and institutional social media (Twitter, Instagram, and Facebook). Prior to participation, participants were sent an information kit containing Participant Information Statement, consent form and resources to support constructive, respectful dialogue. Participants were reimbursed via $30 Prezzee gift vouchers.

## ***Analysis***

Post transcription, two youth CPRT members, conducted the qualitative analysis of data by coding transcripts into themes and sub themes and identifying key quotes. Youth CPRT members were provided training to build capacity in qualitative coding and thematic analysis. There was an open-door ethos of access for the youth CPRT members to contact a research mentor on the CPRT at any time during the coding process. These meetings provided an opportunity for the youth CPRT members to communicate any questions or concerns and to seek clarification. Youth CPRT members collated their findings which consisted of the main themes from the transcript/s, presented in Table 4.

## **Stage 3: YAB consensus workshop**

A structured consensus workshop was conducted to identify and prioritise key principles and priorities for mental health and substance use prevention research and, based on these principles and priorities to develop a list of recommended actions that governments can take to mitigate the mental health impacts of COVID-19 on young people.

The workshop was divided into two sections. In the first section, members of the CPRT delivered a structured PowerPoint presentation summarising key findings from the Stage 1 survey, along with major themes and illustrative quotes from the Stage 2 focus groups. In the second section, the online collaboration tool EasyRetro (<https://easyretro.io/>) was used to facilitate discussion, document ideas, and achieve consensus. CPRT moderators guided a structured group discussion, instructing participants to engage with the digital tool. The EasyRetro board was pre-configured with three columns: **Principles, Priorities, and Actions**. YAB members were asked to input their ideas into the respective columns, after which the group collectively discussed and refined the content until consensus was reached.

## ***Participants***

Participants eligible for Stage 3 were current YAB members including youth CPRT members. Prior to the workshop, YAB participants were sent an information kit containing the participant information statement, consent form and resources to support constructive and respectful dialogue. They were also provided a written summary of Stage 1 and 2 results, workshop aims, participant instructions and key questions for discussion. Participants were reimbursed in accordance with Centre Guidelines on Youth Participation^33^. The workshop was designed by youth CPRT team members and at their request facilitated by a research CPRT member to allow full participation by youth CPRT team members.
