# Supplementary material for: The Coproduced Youth Priorities Project: Australian Youth Priorities for Mental Health and Substance Use Prevention Research
Source: Health Expect. 2025 Apr 23;28(3):e70274. doi: 10.1111/hex.70274 (PMC12015976; doi:10.1111/hex.70274)
Supplement: Supplementary file 2 — Supplementary_material_B_Stage_1_Online_Survey_Questions. [file HEX-28-e70274-s001.docx]

**Youth Priorities for Mental Health and Substance Use Research Survey**

**Note:*

- Indicates participant can choose multiple responses
- Indicates participant can choose only one response

This survey will take approximately 30 minutes to complete.

This survey aims to understand perspectives of Australian youth on priorities for prevention research in mental health and substance use (i.e., alcohol and other drug use). You will be asked questions about yourself, and your views on important topics related to youth involvement in priority-setting for mental health and substance use research and issues affecting youth mental health in Australia.

Your feedback will be used to inform future research and policy submissions.

Instructions:

1. Read each question carefully
2. Please try to answer all questions
3. It is important that you answer each question as honestly as possible. There are no right or wrong answers

**1.** What is your age? (in years) _____________________

(forced to give a numeric value between 16 and 25)

**2.**  How do you describe your gender?

- Man or Male
- Woman or Female
- Non-binary
- I use a different term (please specify) _______________
- Prefer not to answer

**3.** How do you describe your sexual orientation?

- - Straight (heterosexual)
  - Gay or Lesbian
  - Bisexual
  - I use a different term (please specify, optional open text)
  - Don’t know
  - Prefer not to answer

**4.** In which Australian state/territory do you currently live?

- New South Wales
- Victoria
- Queensland
- Northern Territory
- South Australia
- Western Australia
- Tasmania
- Australian Capital Territory

**5.** Which best describes the area where you live?

- Metropolitan
- Regional
- Rural / Remote

**6.** In which country were you born?

- Australia
- Other (please specify)

**7.** Do you have lived experience of a mental health condition/s?

- Yes
- No
- Prefer not to say

**8.** Do you care for a family member or a friend who has lived experience of a mental health condition/s?

- Yes
- No
- Prefer not to say

**9.** Do you have lived experience of an alcohol or other drug use issue/s?

- Yes
- No
- Prefer not to say

**10.** Do you care for a family member or a friend who has lived experience of an alcohol or other drug use issue/s?

- Yes
- No
- Prefer not to say

**What is mental health and substance use prevention research?**

**Prevention programs** aim to reduce the occurrence or progression of mental health and substance use disorders.

**Prevention research** aims to develop, evaluate, and implement innovative approaches to prevention in schools and communities to reduce the impact and cost of mental health and substance use disorders.

**11.** Please rate your agreement or disagreement with the following statement. Young people should be involved in setting the national mental health and substance use prevention research and policy agenda (by this we mean young people should have a say in setting priorities for research and policy related to young people)

- Strongly agree
- Agree
- Disagree
- Strongly disagree
- Comment (optional open text)

Below are a number of ways young people can be involved in setting research priorities. Please rate how useful you consider each activity to be.

**12.** Attending priority setting workshops with other young people

- Extremely Useful
- Somewhat Useful
- Not very useful
- Not useful at all

**13.** Completing priority setting surveys about youth priority issues

- - - Extremely Useful
    - Somewhat Useful
    - Not very useful
    - Not useful at all

**14.** Being a member a Youth Advisory Board/Youth Reference Group for a research organisation

- Extremely Useful
- Somewhat Useful
- Not very useful
- Not useful at all

**15.** Personally sharing social Media activity/posts about important issues

- Extremely Useful
- Somewhat Useful
- Not very useful
- Not useful at all

**16.** Contributing to policy and reform (e.g., as members of advisory groups, meetings with members of local, state or territory governments)

- - - Extremely Useful
    - Somewhat Useful
    - Not very useful
    - Not useful at all
    - Other policy and reform activities (optional open text)

**17.** Currently only a very small proportion of the total amount of government funding that goes to mental health is directed towards ***preventing*** the onset of mental health and substance use disorders. Do you think increased investment in prevention research important? (optional open text)

**18.** The mental health of young people has been significantly impacted by the COVID‑19 pandemic. What support should governments provide reduce these mental health impacts? (optional open text)

**19.** The following question asks you to review the following list of priority populations for targeting mental health and substance use prevention research and rank importance of each on a scale of

1 to 4

| **Priority population** | **Not important at all**  **1** | **Somewhat unimportant**  **2** | **Somewhat important**  **3** | **Very important**  **4** |
| --- | --- | --- | --- | --- |
| Aboriginal and Torres Strait Islander people | 1 | 2 | 3 | 4 |
| People experiencing socio-economic disadvantage | 1 | 2 | 3 | 4 |
| People who have experienced traumatic or stressful events (e.g., physical or sexual assault, accidents, natural disasters) | 1 | 2 | 3 | 4 |
| Young people | 1 | 2 | 3 | 4 |
| Older people | 1 | 2 | 3 | 4 |
| People in contact with the criminal justice system | 1 | 2 | 3 | 4 |
| Culturally and linguistically diverse populations | 1 | 2 | 3 | 4 |
| People who live in regional, rural or remote areas | 1 | 2 | 3 | 4 |
| People identifying as lesbian, gay, bisexual, transgender, or intersex | 1 | 2 | 3 | 4 |

**20.** The following question asks you to review a shorter list of priority populations and select **one** that you think is the **most important**

If you are unsure, you can choose not to rate them by selecting 'no opinion'

| **Priority population** |
| --- |
| Aboriginal and Torres Strait Islander people |
| Those experiencing socio-economic disadvantage |
| Young people |
| Older people |
| People in contact with the criminal justice system |
| Culturally and linguistically diverse populations |
| People who live in regional, rural or remote areas |
| People identifying as lesbian, gay, bisexual, transgender, or intersex |
| Other (please specify, optional open text) |
| No opinion |

**21.** How important is it for mental health prevention research to consider these other behaviours?

| **Co-occurring factors** | **Not important at all**  **1** | **Somewhat unimportant**  **2** | **Somewhat important**  **3** | **Very important**  **4** |
| --- | --- | --- | --- | --- |
| Alcohol use | 1 | 2 | 3 | 4 |
| Tobacco use  (including vaping) | 1 | 2 | 3 | 4 |
| Other drug use | 1 | 2 | 3 | 4 |
| Screen time | 1 | 2 | 3 | 4 |
| Fruit and vegetable consumption | 1 | 2 | 3 | 4 |
| Physical activity | 1 | 2 | 3 | 4 |
| Sleep | 1 | 2 | 3 | 4 |

**22.** Thinking about these behaviours, which is the **most important** for future mental health prevention research?

| **Co-occurring factors** |
| --- |
| Alcohol use |
| Tobacco use (including vaping) |
| Other drug use |
| Recreational screen time |
| Fruit and vegetable consumption |
| Physical activity |
| Sleep |
| Other (please specify) |
| No opinion |

**23.** How important is it to study these behaviours together (i.e., occurring together, as opposed to behaviours that occur separately to mental health)?

- - Not at all important
  - Somewhat important
  - Somewhat important
  - Very important

Why/why not (optional open text)

**24.** Childhood and young adulthood (i.e., ages 0-25) is a period where multiple factors might have a combined impact on a person’s mental health. These factors are often called ‘social determinants’. How important is it to consider the following social determinant factors in mental health prevention research?

| **Social determinant factors** | **Not important at all**  **1** | **Somewhat unimportant**  **2** | **Somewhat important**  **3** | **Very important**  **4** |
| --- | --- | --- | --- | --- |
| Unemployment, precarious employment, and poor employment conditions | 1 | 2 | 3 | 4 |
| Low income | 1 | 2 | 3 | 4 |
| Discrimination related to race/ethnicity, immigrant status, sexual orientation, and/or occupational status | 1 | 2 | 3 | 4 |
| Family relationships | 1 | 2 | 3 | 4 |
| Neighbourhood safety | 1 | 2 | 3 | 4 |
| Secure housing | 1 | 2 | 3 | 4 |
| Food security | 1 | 2 | 3 | 4 |

**25.** Thinking about these social determinant factors, which is the **most important** for future research?

| **Social determinants of mental health** |
| --- |
| Unemployment, precarious employment, and poor employment conditions |
| Low income |
| Discrimination related to race/ethnicity, immigrant status, sexual orientation, and/or occupational status |
| Access to education and skill development |
| Family relationships |
| Neighbourhood safety |
| Secure housing |
| Food security |

**26.** How important is it to study the combined impact of these social determinants together on mental health, rather than each social determinant alone? (For example, how important would it be to study how low-income and unemployment *together* impact youth mental health, rather than studying the singular impact of low-income on mental health)?

- - Not at all important
  - Somewhat important
  - Somewhat important
  - Very important
  - Unsure

Why/why not (optional open text)

**27.** The PREMISE Youth Advisory Board involved in designing this survey has identified 10 broad issues negatively impacting youth mental health in Australia.

The following question asks you to review a shorter list of priorities and select the top one that you think is the most important and relevant to young people's mental health.

If you are unsure of the importance of any of the below-mentioned priorities, you can choose not to rate them by selecting 'no opinion'

| **Most important issues affecting youth mental health** |
| --- |
| Drugs and alcohol |
| Climate Change |
| COVID-19 |
| Unemployment |
| Poverty |
| Intergenerational trauma |
| Unhealthy relationships |
| Isolation and social disconnectedness |
| Lack of accessibility to healthcare in rural and remote settings |
| Social inequality |
| Other (please specify, optional open text) |
| No opinion |

**28.** We would like to involve young people in future research to understand youth priorities for mental health and substance use prevention research. Please select below which format of future research you would prefer to participate in (optional)

1. Completing another survey about youth priorities
2. Participating in a workshop about youth priorities

End of survey

Click ‘submit’ to finish the survey and not enter the prize draw.

If you would like to enter the prize draw, select the ‘Click here to enter draw’ option below.

**For those who do want to enter the draw:**

If you would like to enter the draw to win one of two $250 Prezzie vouchers, please provide your email address: ____________________________________ (free text)

**Click here to Enter Draw**

**Final page (for people who don’t enter draw):**

Thank you for completing this survey. Your responses have been recorded. The information you have provided is very important to us and will be extremely valuable in helping understand and respond to youth priorities for mental health and substance use prevention research.

**If any of the questions raised in this survey have made you feel distressed, please feel free to contact the following services for confidential advice with any issues you may want to discuss.**

| **Name/Organisation** | Lifeline Australia |
| --- | --- |
| **Telephone** | 13 11 14 |
| **Text** | 0477 13 11 14 |

| **Name/Organisation** | Kids Helpline |
| --- | --- |
| **Telephone** | 1800 55 1800 24 |
| **Email** | [counsellor@kidshelpline.com.au](https://kidshelpline.com.au/get-help/email-counselling/) |

**Final page (for people who enter draw):**

Thank you! You have successfully been entered into the draw to win one of two $250 prezzie vouchers. This competition will close (DATE/TIME) and the winner will be notified via email, no later than (INSERT DATE)

Thank you for completing this survey. Your responses have been recorded. The information you have provided is very important to us and will be extremely valuable in in helping understand and respond to youth priorities for mental health and substance use prevention research

**If any of the questions raised in this survey have made you feel distressed, please feel free to contact the following services for confidential advice with any issues you may want to discuss.**

| **Name/Organisation** | Lifeline Australia |
| --- | --- |
| **Telephone** | 13 11 14 |
| **Text** | 0477 13 11 14 |

| **Name/Organisation** | Kids Helpline |
| --- | --- |
| **Telephone** | 1800 55 1800 24 |
| **Email** | [counsellor@kidshelpline.com.au](https://kidshelpline.com.au/get-help/email-counselling/) |
